# Application and evaluation of NCCN guidelines in health education for lung nodule screening: A perspective

**DOI:** 10.1097/MD.0000000000041798

**Published:** 2025-03-14

**Authors:** Chen-Chen Wang, Jian Zhou, Xue Zhao, Xue Gao, Feng-Hua Wang, Ping Bu, Yu-Feng Li

**Affiliations:** a Second Ward of the Department of General Surgery, Hongqi Hospital Affiliated to Mudanjiang Medical University, Mudanjiang, China; b Department of Physical Examination, Hongqi Hospital Affiliated to Mudanjiang Medical University, Mudanjiang, China; c Department of Otorhinolaryngology, Hongqi Hospital Affiliated to Mudanjiang Medical University, Mudanjiang, China; d Department of Thoracic Surgery, Hongqi Hospital Affiliated to Mudanjiang Medical University, Mudanjiang, China.

**Keywords:** application, evaluation, health education, lung nodule, National Comprehensive Cancer Network

## Abstract

This study investigates the application and evaluation of National Comprehensive Cancer Network (NCCN) guidelines within health education frameworks aimed at lung nodule screening. Through the integration of NCCN directives, tailored educational strategies catering to diverse demographics, and robust interdisciplinary collaboration, the research underscores the pivotal role of health education in optimizing screening efficacy and patient outcomes. Moreover, it critically analyzes the challenges encountered, offering insightful recommendations for future research and practice while avoiding replication of existing literature. This study contributes to the field with scholarly rigor, emphasizing the imperative of continuous education in improving patient care standards and mitigating the burden of lung cancer.

## 1. Introduction

Lung nodules pose a significant health risk due to their potential association with lung cancer.^[1]^ These small, round or oval-shaped growths can be benign or malignant. Advances in imaging technology have led to an increase in incidental findings.^[2]^ Early detection of lung nodules is critical as it can substantially improve patient outcomes and reduce mortality rates associated with lung cancer.^[2]^

The National Comprehensive Cancer Network (NCCN) guidelines serve as essential directives for healthcare professionals in managing lung nodules.^[3]^ These guidelines provide evidence-based recommendations for screening, evaluation, and managing lung nodules, standardizing care, and improving patient outcomes.^[3,4]^

Health education plays a crucial role in ensuring patient comprehension of the importance of lung nodule screening and adherence to recommended protocols.^[5]^ By providing clear information about the risks and benefits of screening and empowering patients to make informed health decisions, health education enhances understanding and compliance with screening guidelines.^[5]^

This study aims to highlight the indispensable role of health education in promoting effective lung nodule screening practices and improving patient outcomes. By identifying strategies to improve health education delivery and evaluating its impact on patient behavior and outcomes, this research contributes to the ongoing efforts to reduce the burden of lung cancer through early detection and intervention.

## 2. Application of NCCN guidelines in health education

### 2.1. Educational content and delivery

Integrating the key components of the NCCN guidelines into educational materials ensures the dissemination of accurate and current information concerning lung nodule screening.^[3]^ These materials typically cover crucial topics, including the definition of lung nodules, risk factors for lung cancer, the significance of early detection, and the recommended screening protocols outlined by the NCCN guidelines.^[3]^ Additionally, detailed explanations of imaging modalities, such as computed tomography scans, are provided to enhance patient comprehension.^[6]^

Various methodologies are employed to deliver this education to patients, catering to diverse learning preferences and accessibility needs. Conventional approaches encompass printed brochures distributed within healthcare settings, informational seminars led by healthcare professionals, and individual counseling sessions. Furthermore, digital media platforms, such as websites, mobile applications, and educational videos, increasingly reach a broader audience and facilitate self-directed learning.^[7,8]^ These multimedia resources afford patients the convenience of accessing information remotely while reinforcing key concepts through interactive elements.^[7,8]^

### 2.2. Adaptations for diverse populations

Educational strategies are meticulously tailored to address the distinct needs of diverse demographic groups, encompassing individuals from varying cultural backgrounds, age cohorts, and levels of health literacy.^[9]^ Although educational strategies are tailored for diverse populations, certain barriers may still impede the effectiveness of these interventions. Key challenges include linguistic differences, culturally ingrained beliefs, and disparities in health literacy levels, particularly in settings characterized by multicultural or multilingual populations. Overcoming these obstacles requires targeted communication strategies designed to foster meaningful patient engagement and improve comprehension across diverse groups. Culturally sensitive materials are curated to acknowledge cultural beliefs, language preferences, and socio-economic considerations that may influence patient engagement with educational content.^[8,9]^ For instance, translating educational materials into multiple languages and using visual aids, such as illustrations and diagrams, are instrumental in overcoming language barriers and enhancing comprehension.^[8,9]^

Moreover, educational interventions are adapted to accommodate differing levels of health literacy and numeracy among patients.^[10]^ Using simplified language, plain terminology, and easily comprehensible visuals helps effectively convey complex medical concepts.^[10]^ Additionally, interactive activities, such as quizzes and role-playing scenarios, are integrated into educational sessions to foster active participation and reinforce learning outcomes.

### 2.3. Interdisciplinary collaboration

Interdisciplinary collaboration among healthcare teams is imperative for disseminating information in alignment with NCCN guidelines and ensuring holistic patient care.^[3]^ Pulmonologists, oncologists, nurses, and other healthcare professionals collaborate closely to deliver consistent and coherent messaging regarding lung nodule screening. Each member of the healthcare team assumes a unique role in educating patients and addressing their individual concerns and preferences.^[1–14]^

Pulmonologists and oncologists serve as primary sources of medical expertise, providing patients with comprehensive explanations of screening results and recommendations derived from the NCCN guidelines.^[3]^ Nurses play a pivotal role in delivering education during clinical encounters, offering guidance on scheduling screening appointments, elucidating procedural intricacies, and providing ongoing support throughout the screening process. Moreover, ancillary staff, such as medical assistants and patient navigators, aid in care coordination and facilitate communication between patients and healthcare providers.

By harnessing the collective expertise of interdisciplinary healthcare teams, educational initiatives effectively disseminate information aligned with NCCN guidelines, augment patient understanding, and promote adherence to recommended screening protocols.^[11–14]^ This collaborative approach underscores the principles of patient-centered care and contributes to enhanced outcomes in lung nodule screening and lung cancer management.

## 3. Evaluation of educational outcomes

### 3.1. Impact on patient knowledge

Numerous studies have rigorously examined the effectiveness of educational interventions in augmenting patient knowledge regarding lung nodules and the significance of screening.^[15]^ Typically employing pre- and post-education assessments, these studies carefully evaluate shifts in patient understanding. Pre-education assessments serve to identify existing knowledge gaps, while post-education assessments gauge the extent to which educational interventions have successfully addressed these deficiencies.

Consistently, research findings demonstrate a notable enhancement in patient knowledge subsequent to educational interventions.^[15,16]^ Patients exhibit a heightened comprehension of key concepts such as the definition of lung nodules, associated risk factors for lung cancer, and the rationale behind early detection through screening.^[16]^ Moreover, heightened awareness regarding the benefits of screening and the potential consequences of undetected lung nodules is consistently observed post-education. By providing patients with accurate information, educational interventions facilitate informed decision-making and foster proactive engagement in healthcare management.

### 3.2. Compliance with screening protocols

Education founded on NCCN guidelines assumes a critical role in cultivating patient adherence to recommended screening protocols.^[17–19]^ By explaining the importance of regular screening and the potential implications for early detection and treatment outcomes, educational interventions motivate patients to prioritize their health and adhere to screening schedules.^[17]^ Studies scrutinizing the impact of education on patient compliance consistently report elevated rates of adherence among individuals who have undergone educational interventions compared to their counterparts.^[17,18]^

Furthermore, personalized education tailored to the distinct needs of diverse demographic groups enhances patient engagement and instills a sense of accountability towards screening. Culturally sensitive and linguistically appropriate educational materials resonate with patients from various backgrounds, fostering trust and facilitating effective communication with healthcare providers.^[19]^ Consequently, patients are more inclined to honor screening appointments and adhere to recommended follow-up protocols, thereby optimizing the efficacy of lung nodule screening programs.

### 3.3. Screening results

The association between enhanced education and early detection of lung nodules remains a subject of ongoing investigation. Preliminary evidence suggests that improved patient education contributes to the expedited identification of lung nodules and favorable management outcomes.^[13]^ Well-informed patients are more vigilant in recognizing suspicious symptoms or abnormalities detected on imaging studies, thereby facilitating prompt medical intervention.

Moreover, education empowers patients to actively engage in shared decision-making regarding their healthcare management. Informed patients are more inclined to collaborate with healthcare providers in devising personalized screening plans tailored to their individual risk profiles and preferences.^[20,21]^ This patient-centered approach facilitates comprehensive evaluation and management of lung nodules, culminating in improved clinical outcomes and heightened patient satisfaction.

In essence, educational interventions grounded in NCCN guidelines exert a profound influence on patient knowledge, compliance with screening protocols, and screening outcomes. By equipping patients with essential information and resources to make informed decisions about their health, educational initiatives significantly contribute to the early detection and effective management of lung nodules, thereby mitigating the burden of lung cancer and enhancing patient outcomes.^[20,21]^

## 4. Challenges and limitations

### 4.1. Barriers in implementation

Despite the recognized benefits of incorporating NCCN guidelines into routine patient education, several obstacles impede their seamless integration into healthcare practices. Language barriers, particularly prevalent in multicultural societies, pose a significant challenge. Patients with limited proficiency in the primary language of healthcare settings may struggle to understand educational materials, requiring translation efforts that demand additional time and resources for providers.^[22]^

Resource limitations also hinder the widespread adoption of comprehensive educational programs. Many healthcare facilities face constraints in funding and infrastructure, limiting their capacity to develop and disseminate educational materials effectively. Implementing NCCN guidelines within health education programs presents considerable challenges, particularly in underfunded or resource-constrained healthcare environments. Key barriers include limited availability of trained personnel, restricted financial resources, and inadequate infrastructure, all of which hinder the scalability and broader adoption of these guidelines.^[23,24]^ Moreover, staffing shortages and competing priorities in healthcare settings may limit personnel availability to deliver consistent educational interventions, complicating implementation efforts.^[24]^

Additionally, patient reluctance presents a formidable barrier to successful implementation.^[24]^ Despite educational efforts, some individuals may harbor misconceptions or fears regarding lung nodule screening, leading to hesitancy or refusal to participate. Overcoming these barriers demands tailored communication strategies that address individual concerns and foster trust between patients and healthcare providers.

### 4.2. Limitations of current studies

While existing research has yielded valuable insights into the impact of educational interventions on patient outcomes, several limitations undermine the robustness of the evidence base.^[25–28]^ Persistent gaps in the literature, particularly regarding long-term follow-up and sustainability of educational effects, challenge the validity and generalizability of findings. Many studies rely on short-term outcome measures, such as changes in patient knowledge or immediate compliance with screening protocols, without assessing the durability of these effects over time.

Furthermore, potential biases inherent in existing research may compromise the validity of conclusions drawn.^[25,26]^ Selection bias, for instance, may arise if studies predominantly recruit participants from specific demographic groups or healthcare settings, limiting the applicability of results to broader populations. Similarly, publication bias may skew the literature towards studies reporting positive outcomes, while studies with null or negative findings may remain unpublished or under-reported.^[25]^

Methodological limitations, such as small sample sizes, lack of control groups, and reliance on self-reported measures, further undermine the reliability of study findings.^[26,27]^ These limitations impede the ability to draw definitive conclusions about the effectiveness of educational interventions in real-world clinical settings.

Addressing these challenges and limitations necessitates collaborative efforts from healthcare providers, researchers, and policymakers. Culturally competent approaches, strategic resources allocation, and methodological rigor are essential for enhancing the effectiveness and sustainability of educational initiatives aimed at improving lung nodule screening practices and patient outcomes.^[28]^ Moreover, ongoing evaluation and refinement of educational programs are critical to ensuring their continued relevance and impact amidst the evolving landscape of healthcare delivery.

## 5. Future directions

### 5.1. Recommendations for practice

Healthcare providers should prioritize the development of culturally sensitive and linguistically tailored educational materials to optimize the application of NCCN guidelines in patient education programs.^[29,30]^ Adopting interactive and multimedia educational approaches, such as mobile applications and virtual reality simulations, can enhance educational effectiveness.^[29]^ Additionally, fostering interdisciplinary collaboration among healthcare teams is imperative for delivering consistent and cohesive messaging regarding lung nodule screening.

### 5.2. Research opportunities

Longitudinal studies assessing the sustained impact of educational interventions on patient knowledge, behavior, and health outcomes are essential.^[31]^ Exploring the effectiveness of novel educational strategies, such as peer-led interventions and community-based initiatives, offers promising research opportunities. Comparative effectiveness research on various educational approaches can inform evidence-based practice guidelines. Furthermore, research addressing barriers to implementation, such as language barriers and resource limitations, is vital for enhancing programmatic effectiveness and sustainability.^[31]^

## 6. Summary

In summary, the application and evaluation of NCCN guidelines within lung nodule screening contexts have yielded valuable insights, shaping the landscape of health education practices. Through meticulous application of these guidelines, healthcare providers have witnessed tangible improvements in patient understanding and adherence to screening protocols.

The implications for health education practice are profound. Structured patient education emerges as a cornerstone in the effective dissemination of critical information regarding lung nodule screening. By integrating NCCN guidelines into educational materials and fostering interdisciplinary collaboration among healthcare teams, providers can optimize patient comprehension and engagement.

Looking ahead, the importance of ongoing education and guideline updates cannot be overstated. As healthcare practices evolve alongside technological advancements, the need for continuous education remains paramount. Staying abreast of updated guidelines ensures that patients receive accurate, culturally sensitive information, thus contributing to the early detection and management of lung nodules. This commitment to continuing education underscores the dedication of healthcare providers to enhancing patient care and mitigating the burden of lung cancer.

## Acknowledgments

This study was partly supported by The Basic Research Fund Project of Provincial Universities of Heilongjiang Province (2020-KYYWFMY-0027).

## Author contributions

**Conceptualization:** Chen-Chen Wang, Feng-Hua Wang, Yu-Feng Li.

**Data curation:** Chen-Chen Wang, Xue Gao, Ping Bu, Yu-Feng Li.

**Investigation:** Yu-Feng Li.

**Methodology:** Jian Zhou, Feng-Hua Wang, Ping Bu, Yu-Feng Li.

**Project administration:** Yu-Feng Li.

**Resources:** Chen-Chen Wang, Jian Zhou, Xue Zhao, Xue Gao, Feng-Hua Wang, Ping Bu.

**Supervision:** Yu-Feng Li.

**Validation:** Chen-Chen Wang, Jian Zhou, Xue Zhao, Xue Gao, Feng-Hua Wang, Ping Bu, Yu-Feng Li.

**Visualization:** Chen-Chen Wang, Jian Zhou, Xue Zhao, Xue Gao, Feng-Hua Wang, Ping Bu, Yu-Feng Li.

**Writing – original draft:** Chen-Chen Wang, Jian Zhou, Xue Gao, Feng-Hua Wang, Ping Bu, Yu-Feng Li.

**Writing – review & editing:** Chen-Chen Wang, Jian Zhou, Xue Zhao, Xue Gao, Feng-Hua Wang, Ping Bu, Yu-Feng Li.
